# Cemiplimab in locally advanced or metastatic cutaneous squamous cell carcinoma: prospective real-world data from the DRUG Access Protocol

**DOI:** 10.1016/j.lanepe.2024.100875

**Published:** 2024-03-05

**Authors:** Karlijn Verkerk, Birgit S. Geurts, Laurien J. Zeverijn, Vincent van der Noort, Henk M.W. Verheul, John B.A.G. Haanen, Astrid A.M. van der Veldt, Ferry A.L.M. Eskens, Maureen J.B. Aarts, Carla M.L. van Herpen, Mathilde Jalving, Jourik A. Gietema, Lot A. Devriese, Mariette Labots, Sahar Barjesteh van Waalwijk van Doorn-Khosrovani, Egbert F. Smit, Haiko J. Bloemendal

**Affiliations:** a Division of Molecular Oncology & Immunology, Netherlands Cancer Institute, Amsterdam, the Netherlands; b Oncode Institute, Utrecht, the Netherlands; c Department of Biometrics, Netherlands Cancer Institute, Amsterdam, the Netherlands; d Department of Medical Oncology, Erasmus Medical Center, Rotterdam, the Netherlands; e Department of Medical Oncology, Netherlands Cancer Institute, Amsterdam, the Netherlands; f Department of Clinical Oncology, LUMC, Leiden, the Netherlands; g Head of Melanoma Clinic, CHUV, Lausanne, Switzerland; h Department of Radiology & Nuclear Medicine, Erasmus Medical Center, Rotterdam, the Netherlands; i Department of Medical Oncology, GROW School for Oncology and Developmental Biology, Maastricht University Medical Center, Maastricht, the Netherlands; j Department of Medical Oncology, Radboud University Medical Center, Nijmegen, the Netherlands; k Department of Medical Oncology, University of Medical Center Groningen, Groningen, the Netherlands; l Department of Medical Oncology, Division Beeld & Oncologie, Utrecht University Medical Center, Utrecht, the Netherlands; m Department of Medical Oncology, Amsterdam University Medical Center, Location VUMC, Cancer Center Amsterdam, the Netherlands; n CZ Health Insurance, Tilburg, the Netherlands; o Department of Medical Oncology, Leiden University Medical Center, Leiden, the Netherlands; p Department of Pulmonology, Leiden University Medical Center, Leiden, the Netherlands

**Keywords:** Cemiplimab, Cutaneous squamous cell carcinoma, Immune checkpoint blockade, Real-world data

## Abstract

**Background:**

The DRUG Access Protocol provides patients with cancer access to registered anti-cancer drugs that are awaiting reimbursement in the Netherlands and simultaneously collects prospective real-world data (RWD). Here, we present RWD from PD-1 blocker cemiplimab in patients with locally advanced or metastatic cutaneous squamous cell carcinoma (laCSCC; mCSCC).

**Methods:**

Patients with laCSCC or mCSCC received cemiplimab 350 mg fixed dose every three weeks. Primary endpoints were objective clinical benefit rate (CBR), defined as objective response (OR) or stable disease (SD) at 16 weeks, physician-assessed CBR, defined as clinician’s documentation of improved disease or SD based on evaluation of all available clinical parameters at 16 weeks, objective response rate (ORR), and safety, defined as grade ≥ 3 treatment related adverse events (TRAEs) occurring up to 30 days after last drug administration. Secondary endpoints included duration of response (DoR), progression-free survival (PFS), and overall survival (OS).

**Findings:**

Between February 2021 and December 2022, 151 patients started treatment. Objective and physician-assessed CBR were 54.3% (95% CI, 46.0–62.4) and 59.6% (95% CI, 51.3–67.5), respectively. ORR was 35.1% (95% CI, 27.5–43.3). After a median follow-up of 15.2 months, median DoR was not reached. Median PFS and OS were 12.2 (95% CI, 7.0-not reached) and 24.2 months (95% CI, 18.8-not reached), respectively. Sixty-eight TRAEs occurred in 29.8% of patients. Most commonly reported TRAE was a kidney transplant rejection (9.5%).

**Interpretation:**

Cemiplimab proved highly effective and safe in this real-world cohort of patients with laCSCC or mCSCC, confirming its therapeutic value in the treatment of advanced CSCC in daily clinical practice.

**Funding:**

The DRUG Access Protocol is supported by all participating pharmaceutical companies: 10.13039/100004326Bayer, Janssen, Lilly, 10.13039/100004334Merck, 10.13039/100004336Novartis, 10.13039/100004337Roche, and 10.13039/100004339Sanofi.

**Research in context**

**Evidence before this study**

Prior to the initiating of this cohort in the DRUG Access Protocol in January 2021, a review of existing literature and clinical trial databases, including Pubmed and the official websites of the European Medicines Agency and Food and Drug Administration, was conducted. This search, unrestricted by language or publication date, focused on keywords such as “cutaneous squamous cell carcinomas”, and therapy, thereof, “immunotherapy”, “immune checkpoint inhibitors”, “immune checkpoint blockade”. This exploration revealed phase 1 (NCT02383212) and phase 2 trials (NCT0276098, NCT02760498) showing a pooled objective response rate of 46.1% of cemiplimab in patients with locally advanced or metastatic cutaneous squamous cell carcinoma. It was also found that, based on these single-arm trials, the European Medicines Agency granted approval to cemiplimab for the treatment of locally advanced or metastatic cutaneous squamous cell carcinomas in 2019. However, given that single-arm trials are highly susceptible to several forms of bias, including selection bias due to underrepresentation of certain patients populations (e.g., the elderly and patients with relevant comorbidities), the Dutch healthcare authorities raised concerns about the value of the drug to the individual patient. Hence, patients with locally advanced or metastatic squamous cell carcinoma in the Netherlands did not have access to cemiplimab. In summary, the existing clinical data about the efficacy of cemiplimab in patients with locally advanced or metastatic cutaneous squamous cell carcinoma and the gap in patient access to cemiplimab in the Netherlands, established a strong clinical foundation for further investigation of cemiplimab in patients with locally advanced or metastatic squamous cell carcinoma in a real-world setting, to thereby provide access to cemiplimab and facilitate reimbursements evaluations in the Netherlands.

**Added value of this study**

To our knowledge, this is the first prospective protocol reporting on the efficacy and safety of cemiplimab in a real-world cohort of patients with locally advanced or metastatic cutaneous squamous cell carcinoma. After analyzing data from 151 patients that were granted access to cemiplimab through a personalized reimbursement model in the DRUG Access Protocol, our results were in line with those found in the previous single-arm trials. As our population was generally older and included a significant proportion of patients that had characteristics which would render them ineligible for participation in the previous registration trials (e.g., frail performance status, history of auto-immune disease, hematological malignancies, and organ transplants), we could confirm the value of cemiplimab in a real-world setting.

**Implications of all the available evidence**

Our results combined with the existing evidence provided confidence in the value of cempilimab to the individual patient. This resulted in a positive final health technology assessment, leading to reimbursement of cemiplimab in the Netherlands. These results illustrate that real-world data and initiatives like the DRUG Access Protocol have the potential to bridge the gap between drug approval by regulatory agencies and reimbursement on a national level.

## Introduction

Large, phase III, randomized controlled trials are the gold standard to demonstrate efficacy in drug development and approve new treatments. However, this approach is not always feasible in patients with rare cancers or those with tumors harboring rare molecular profiles due to the limited number of patients. Moreover, randomization may be deemed unethical when the investigational drug has shown significant treatment effect in earlier clinical trials.^1^ In these instances, drug approval is reliant on single-arm trials. As these trials are highly susceptible to several forms of bias, including selection bias due to underrepresentation of certain patient populations (e.g., the elderly and patients with comorbidities),^1^ health technology assessment agencies may not directly be convinced by the cost- and clinical effectiveness of these drugs in a real-world setting. Consequently, reimbursement of these drugs is delayed and thus patients do not have access to these new treatment options. To address this issue, the DRUG Access Protocol (DAP) was initiated in February 2021.^2^ This protocol aims to provide patients prompt, controlled access to anti-cancer drugs that are approved by the Food and Drug Administration (FDA) or European Medicines Agency (EMA) but are awaiting reimbursement in the Netherlands, whilst simultaneously collecting prospective real-world data (RWD) on the efficacy and safety of these drugs.

Cutaneous squamous cell carcinoma (CSCC), a malignant proliferation of cutaneous epithelium, is the second most common form of skin cancer.^3^^,^^4^ The incidence in the Netherlands currently is 15.000 cases annually and is rapidly increasing.^5^ Risk factors for CSCC include, amongst others, exposure to ultraviolet (UV) light, immunosuppression, fair skin, and advanced age.^6^^,^^7^ The majority of patients with CSCC (>95%) can be cured with surgery and/or radiotherapy.^8^^,^^9^ However, a small percentage of patients develop locally advanced CSCC (laCSCC) and is not amenable to surgery or radiotherapy, or metastatic CSCC (mCSCC). These patients have incurable disease and a poor long-term prognosis, with relative 5-year survival between 51 and 64%.^10^

Inherent to UV-light induced DNA damage, the median tumor mutational burden of CSCC is high with approximately 45 mutations per megabase.^7^ High tumor mutational burden has been associated with response to immune checkpoint blockade (ICB), including programmed death-1 (PD-1) blockers, likely due to increased neoantigen expression and thereby enhanced immunogenicity.^11^ Furthermore, the strong association between the increased risk of CSCC and immunosuppression indicates that the immune system plays a pivotal role in preventing this malignancy, also suggesting that approaches to enhance antitumor immunity may be effective in treating CSCC.^7^

Over the past few years, sensitivity of laCSCC and mCSCC to ICB has indeed been observed. In phase II single-arm trials, the observed objective response rates (ORR) of the PD-1 blocker cemiplimab in laCSCC and mCSCC were 44% (95% confidence interval (95% CI), 32–55)^7^ and 45.2% (95% CI, 35.9–54.8),^4^ respectively. Pooled disease control rate (DCR) at 15 weeks was 60.6% (95% CI, 53.3–67.6), median progression-free survival (PFS) was 18.4 months (95% CI, 10.3–24.3), and the median duration of response (DoR) and overall survival (OS) were not reached after a median follow-up of 15.7 months, all pointing towards durable responses in a significant proportion of patients.^12^ Furthermore, cemiplimab showed an acceptable safety profile. Based on these data, in 2019 cemiplimab became the first drug receiving approval by the EMA for the treatment of adult patients with laCSCC who are not candidates for curative surgery or curative radiotherapy, or mCSCC.^13^

However, to date, the aforementioned data were considered insufficient to reimburse cemiplimab in the Dutch healthcare system. This is partly based upon the consideration that elderly patients and those with poor performance status or relevant comorbidities (including autoimmune diseases) were underrepresented in these studies. As it is assumed that these subgroups constitute a significant proportion of CSCC patients in daily clinical practice, the real-world effectiveness of cemiplimab remained unclear. To bridge this gap in data, cemiplimab was incorporated in DAP.^2^ Here, we report prospective RWD of the efficacy and safety of cemiplimab in patients with laCSCC or mCSCC treated in DAP.

## Methods

### Protocol design

DAP is a prospective, open-label, non-randomized protocol that collects real-world efficacy and safety data. Patients are enrolled in multiple parallel cohorts, each defined by an approved anti-cancer drug awaiting reimbursement. For this cohort, a pragmatic personalized reimbursement model, similar to the one previously used in precision oncology cohorts in the Netherlands^14^ was employed. The costs of the first 16 weeks of treatment with cemiplimab were covered by the market authorization holder through a rebate to the payers. Subsequently, upon clinical benefit (CB) at 16 weeks, the costs of continued treatment with cemiplimab were reimbursed by the payers while efficacy and safety data collection continued.^2^

DAP was considered not subject to the Dutch Medical Research Involving Human Subjects Act by the Medical Ethical Committee of the Netherlands Cancer Institute in Amsterdam. DAP was approved by the Institutional Review Board of the Netherlands Cancer Institute in Amsterdam (IRBdm20-203). The protocol is conducted in accordance with Good Clinical Practice guidelines and the Declaration of Helsinki’s ethical principles for medical research. Written informed consent was obtained from all included patients. Six sites were assigned by the health care authorities to participate in DAP, and each participating site obtained local approval from their respective Boards of Directors prior to enrollment of patients and submission of patient data.

### Patients

Eligible patients were adults with laCSCC, for whom either curative surgery or curative radiotherapy were not or no longer possible (at the discretion of the treating physician), or mCSCC. Patients were accrued at the participating hospitals throughout the Netherlands. Patients had to be evaluable according to Response Evaluation Criteria in Solid Tumors (RECIST) v1.1^15^ with either measurable disease (presence of quantifiable lesions that qualify as target lesions) or evaluable disease (presence of only non-target lesions), and had, at the discretion of the treating physician, an acceptable organ function. An expert panel on immunotherapy in CSCC was available to advise treating physicians on eligibility and clinical decision-making if needed. All patients that received at least one administration of cemiplimab were included in the efficacy and safety analyses.

### Protocol procedures

Patients were treated with intravenous cemiplimab (350 mg every 3 weeks) until disease progression or unmanageable toxicity. Response evaluations were performed at baseline and, to align with the specified timelines of the personalized reimbursement model, 16 weeks after start of treatment. If the patient continued treatment beyond the first response evaluation, response evaluations were thereafter performed at 12-week intervals. After consultation with the expert panel on immunotherapy in CSCC, it was decided that interruption of treatment could be considered for patients with ongoing benefit after 24 weeks of treatment. For these patients, retreatment in case of radiological or clinical progression was allowed, provided that the patient still met all eligibility criteria.

### Outcomes

Primary endpoints included objective clinical benefit rate (CBR), physician-assessed clinical benefit (CB), best overall response (BOR), ORR, and safety. Objective CB was defined as complete response (CR), partial response (PR), or stable disease (SD) according to RECIST v1.1 measurements at 16 weeks after treatment initiation. Patients with only evaluable disease could solely be assessed based on non-target lesions and could therefore only achieve CR, progressive disease (PD), and non CR/non PD, where non CR/non PD was considered SD for further analyses. Physician-assessed CB was defined as the documentation in a clinician’s note of improved or stable disease, based on the evaluation of all available clinical parameters (e.g., imaging, physical exam, biomarkers, pathology specimen, and patient-reported concerns) present at 16 weeks after treatment initiation. The BOR was defined as the best response according to RECIST v.1.1 measurements recorded from the start of treatment until disease progression. The ORR was defined as the percentage of confirmed responders according to RECIST v1.1 measurements. Safety was measured by the frequency of grade ≥ 3 treatment related adverse events (TRAEs) and serious adverse events (SAEs) occurring up to 30 days after the last administration of drug. All adverse events (AEs) were graded according to Common Terminology Criteria for Adverse Events version 5.0.^16^

Secondary endpoints included DoR, PFS, time to end of CB, and OS. DoR was calculated from the first date response was measured until the first date disease progression was measured or the patient went off study due to progressive disease or death, censoring patients alive without progression at their last tumor measurement. PFS was calculated similarly, but starting from date of treatment initiation. Time to end of CB was calculated from the first day of treatment until the first time the physician thought the patient did no longer experience CB from treatment at response evaluation or the patient went of study with progressive disease or death, censoring patients alive without progression at their last tumor measurement. OS was calculated from the first day of treatment administration to the date of death from any cause, censoring patients who were alive at their last follow-up date.

### Statistical analyses

Patient characteristics, tumor responses, and AEs were summarized using descriptive statistics. Exact 95% CIs of the CBR and ORR were calculated using the Clopper-Pearson method. Associations between CB and baseline characteristics were calculated with the Fisher’s Exact Test (categorical variables), Wilcoxon test (continuous variables), and linear by linear association test (ordinal variables). p values < 0.05 were considered to be statistically significant. Kaplan–Meier methods were used to estimate time on treatment, DoR, PFS, time to end of CB, and OS. Reverse Kaplan–Meier method was used to estimate the duration of follow-up. Data cut-off for this analysis was September 27th 2023. All statistical analyses were performed using R version 4.2.0.

### Role of funding source

The funders of the study had no role in protocol design, data collection, data analysis, data interpretation, or writing of the report.

## Results

### Accrual and patient characteristics

Between February 2021 and December 2022, 176 cases of patients with laCSCC or mCSCC were submitted to the central DAP team for potential treatment with cemiplimab. Of these, 25 patients did not start with treatment, mainly due to ineligibility (8/25, 32.0%). A full overview of accrual and reasons for non-enrollment is provided in Supplementary Fig. S1. In total, 151 patients were found eligible and started treatment with cemiplimab. Baseline characteristics of these patients are presented in Table 1. The median age of patients in this real-world cohort was 78 years [IQR 72–83] and 20/151 patients (13.2%) had an Eastern Cooperative Oncology Group (ECOG) performance score of 2 or higher. Seven patients (4.6%) had kidney transplants and 17 patients (11.3%) had a history of auto-immune disease.

**Table 1:** Baseline characteristics of patients that started treatment.

	Patients n = 151
Tumor type, n (%)	
Squamous cell carcinoma	151 (100.0)
Age (approximately) at consent, median [IQR]	78.0 [72.0, 83.0]
Biological sex, n (%)	
Male	104 (68.9)
Female	47 (31.1)
ECOG performance status, n (%)	
ECOG 0	45 (29.8)
ECOG 1	85 (56.3)
ECOG 2	19 (12.6)
ECOG 3	1 (0.7)
Not Available	1 (0.7)
Primary site, n (%)	
Head or neck	92 (60.9)
Trunk	24 (15.9)
Upper or lower extremities	23 (15.2)
Other^a^	6 (4.0)
More than one site^b^	4 (2.6)
Unknown	2 (1.3)
Type, n (%)	
mCSCC	99 (65.6)
laCSCC	52 (34.4)
Previous systemic treatment lines, n (%)	
0	143 (94.7)
1	8 (5.3)
Previous radiotherapy, n (%)	
Yes	79 (52.3)
No	72 (47.7)
History of autoimmunedisease, n (%)	
No	134 (88.7)
Yes	17 (11.3)
Corticosteroid use, n (%)	
No	140 (92.7)
Yes	11 (7.3)
Other immunosuppressive drugs, n (%)	
No	144 (95.4)
Yes	7 (4.6)
History of organ transplantation, n (%)	
No	144 (95.4)
Yes	7 (4.6)
History of hematological malignancy, n (%)	
No	138 (91.4)
Yes	13 (8.6)

IQR, interquartile range; ECOG, Eastern Cooperative Oncology Group; laCSCC, locally advanced cutaneous squamous cell carcinoma; mCSCC, metastatic cutaneous squamous cell carcinoma.

a Other tumor locations included conjunctiva (n = 2), hand right (n = 1) forefinger right (n = 1) and feet (n = 2).

b More than one site included head, trunk and arms (n = 1), both legs and left arm (n = 1), head and trunk (n = 1), ear, clavicular and scalp (n = 1).

### Response and clinical benefit

**Fig. 1:**
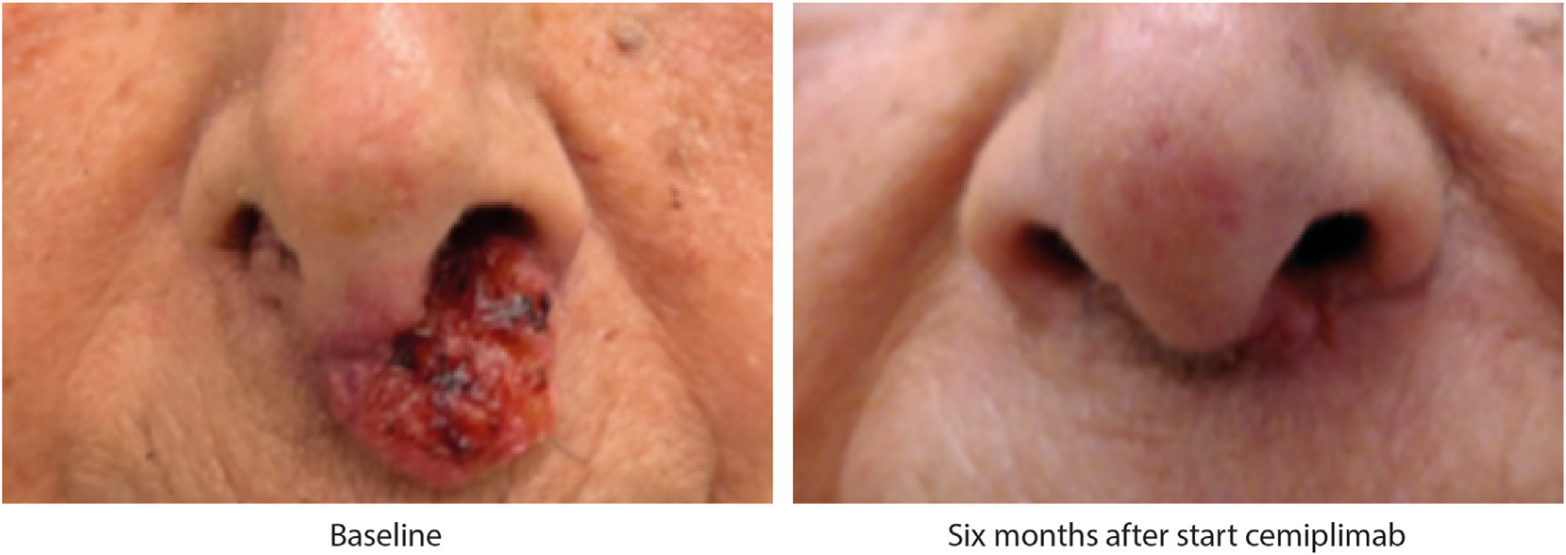
**Effect of cemiplimab in a patient with locally advanced cutaneous squamous cell carcinoma.**

**Fig. 2:**
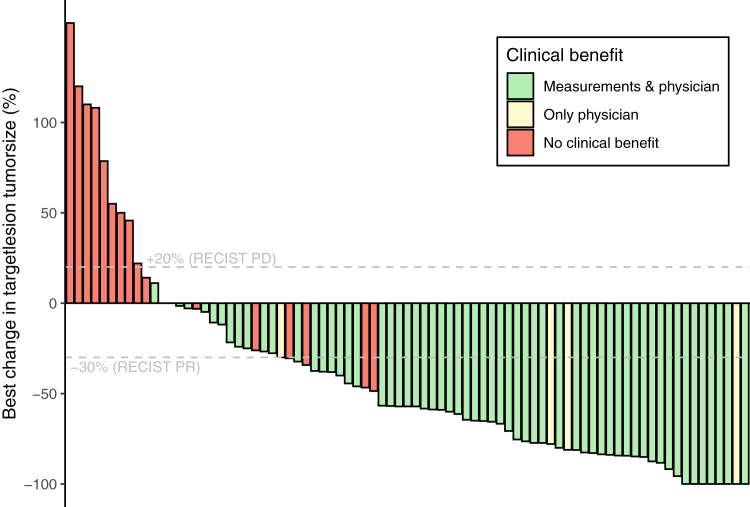
**Waterfall plot depicting best percent change in sum largest diameters.** Legend: Of all included patients in this cohort (n = 151), 118 had measurable disease according to RECIST v.1.1 measurements at baseline. Of those patients, 81 patients also had at least one response assessment where the target lesions could be/were measured. These patients are included in the waterfall plot. Green means the patient had clinical benefit both according to RECIST v.1.1 measurements as well as according to the physician. Yellow indicates that there was no clinical benefit according to RECIST v.1.1 measurements, but there was clinical benefit according to the physician. Red means there was no clinical benefit at all. Abbreviations: PD, progressive disease; PR, partial response, RECIST, Response Evaluation Criteria Solid Tumors.

Of the 151 included patients, 118 (78.1%) patients had measurable disease at baseline while the remaining patients (n = 33, 21.9%) only had evaluable disease. BORs according to RECIST v1.1 criteria are summarized in Table 2, Table 3. In total, 53 patients achieved a confirmed PR or CR, resulting in an ORR of 35.1% (95% CI, 27.5–43.3) when considering all patients. When only considering patients with measurable disease, who could achieve both PR and CR instead of only CR, there were 48 patients with a confirmed PR or CR, resulting in an ORR of 40.7% (95% CI, 31.7–50.1). Of the eight patients with an unconfirmed PR as their BOR, one patient can still achieve a confirmed PR after data cut-off, the remaining seven patients developed PD or deceased. Fig. 1 shows a patient who had a CR after 6 months of treatment. A summary of the best percentage change in sum of target lesions for all patients with measurable disease who had at least one evaluable response evaluation (n = 81) is presented in Fig. 2.

**Table 2:** Best overall response according to RECIST v.1.1 measurements stratified by objective clinical benefit at 16 weeks.

	All patients	Objective clinical benefit	
	n = 151	Clinical benefit n = 82	No clinical benefit n = 69
BOR according to RECIST v.1.1 measurements, n (%)			
CR (confirmed)	18 (11.9)	18 (22.0)	0 (0.0)
CR (unconfirmed)	4 (2.6)	3 (3.7)	1 (1.4)
PR (confirmed)	31 (20.5)	31 (37.8)	0 (0.0)
PR (unconfirmed)	8 (5.3)	6 (7.3)	2 (2.9)
SD ≥ 16 weeks	24 (15.9)	24 (29.3)	0 (0.0)
SD < 16 weeks	4 (2.6)	0 (0.0)	4 (5.8)
PD	42 (27.8)	0 (0.0)	42 (60.9)
NE	20 (13.2)	0 (0.0)	20 (29.0)

BOR, best overall response; CR, complete response; PR, partial response; SD, stable disease; PD, progressive disease; NE, non-evaluable. Patients that had an unconfirmed PR could still have clinical benefit if they did have stable disease for ≥16 weeks.

**Table 3:** Best overall response according to RECIST v.1.1 measurements stratified by physician-assessed clinical benefit at 16 weeks.

	All patients	Physician-assessed clinical benefit	
	n = 151	Clinical benefit n = 90	No clinical benefit n = 61
BOR according to RECIST v.1.1 measurements, n (%)			
CR (confirmed)	18 (11.9)	18 (20.0)	0 (0.0)
CR (unconfirmed)	4 (2.6)	3 (3.3)	1 (1.6)
PR (confirmed)	31 (20.5)	31 (34.4)	0 (0.0)
PR (unconfirmed)	8 (5.3)	7 (7.8)	1 (1.6)
SD ≥ 16 weeks	24 (15.9)	24 (26.7)	0 (0.0)
SD < 16 weeks	4 (2.6)	0 (0.0)	4 (6.6)
PD	42 (27.8)	6 (6.7)	36 (59.0)
NE	20 (13.2)	1 (1.1)	19 (31.1)

BOR, best overall response; CR, complete response; PR, partial response; SD, stable disease; PD, progressive disease; NE, non-evaluable. Patients that had an unconfirmed PR could still have clinical benefit if they did have stable disease for ≥16 weeks.

Eighty-two out of 151 patients had confirmed CR, PR, or SD ≥ 16 weeks, resulting in an objective CBR of 54.3% (95% CI, 46.0–62.4) (Table 2). The number of patients that had physician-assessed CB was 90, resulting in a physician-assessed CBR of 59.6% (95% CI, 51.3–67.5) (Table 3). Lower ECOG performance status was significantly associated with both objective CB and physician-assessed CB (p = 0.043 and p = 0.012, respectively). Auto-immune diseases, use of corticosteroids, including dexamethasone and prednisone, or use of other immunosuppressive drugs did not have a statistically significant impact on both CB and OR. The presence of hematological malignancies was significantly associated with lack of both objective CB and physician-assessed CB (p = 0.022 and p = 0.007, respectively, Supplementary Table S1A–C).

The discrepancy between objective CBR and physician-assessed CBR is caused by eight patients (5.3%) with discordance between RECIST v1.1 measurements and the physician’s assessment. Two patients (25.0%) were not evaluable according to RECIST v.1.1 criteria and could therefore not be classified as having CB adhering to the objective definition, but did have CB according to their physician. The remaining six cases (75.0%) were truly discordant, as these patients developed PD within 16 weeks according to RECIST v.1.1 measurements, but had CB according to their physician. PD was based on newly enlarged lymph nodes in four patients and unequivocal progression of non-target lesions in two patients.

### Duration of response and survival

**Fig. 3:**
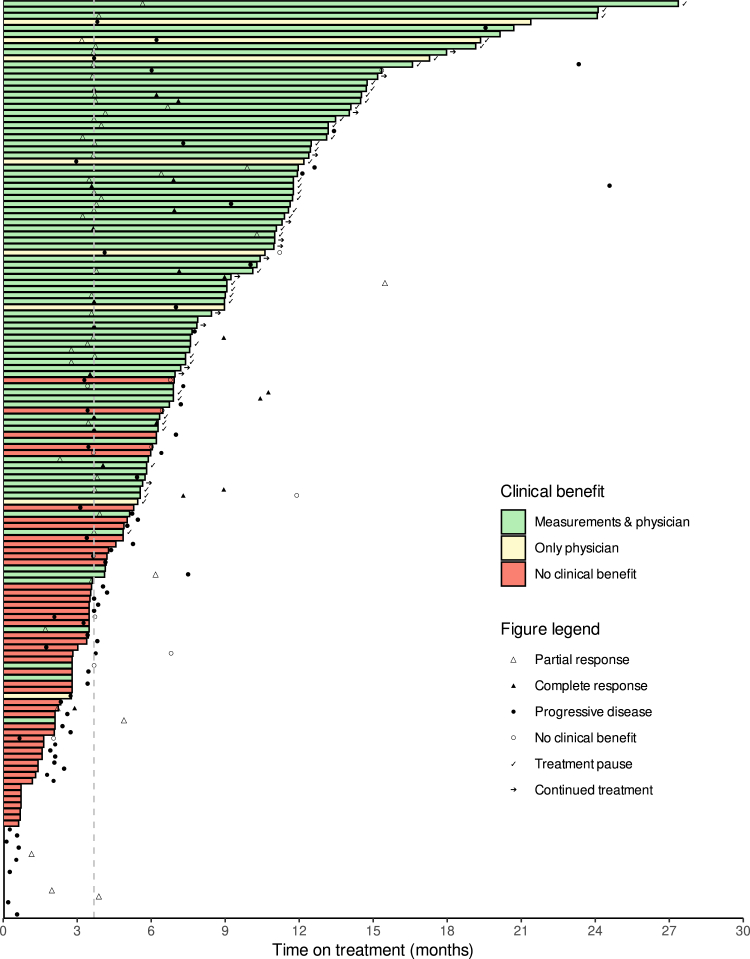
**Swimmer plot.** Legend: Swimmer plot of all patients who started treatment (n = 151). Green means the patient had clinical benefit both according to RECIST v.1.1 measurements as well as according to the physician. Yellow indicates that there was no clinical benefit according to RECIST v.1.1 measurements, but there was clinical benefit according to the physician. Red means there was no clinical benefit at all. Open triangle shapes indicate the first time partial response was measured (regardless of it being confirmed later or not), filled triangle shapes indicate the first time complete response was measured (regardless of it being confirmed later or not). Filled circles indicate the first time progressive disease was measured or there was clinical deterioration related to disease progression. Open circles indicate the first time the physician did not think the patient had benefit from the treatment (often coincides with the progression date). Arrows indicate that the patient is still on treatment and ticks indicate that the patient is on a treatment pause after at least 24 weeks of clinical benefit.

**Fig. 4:**
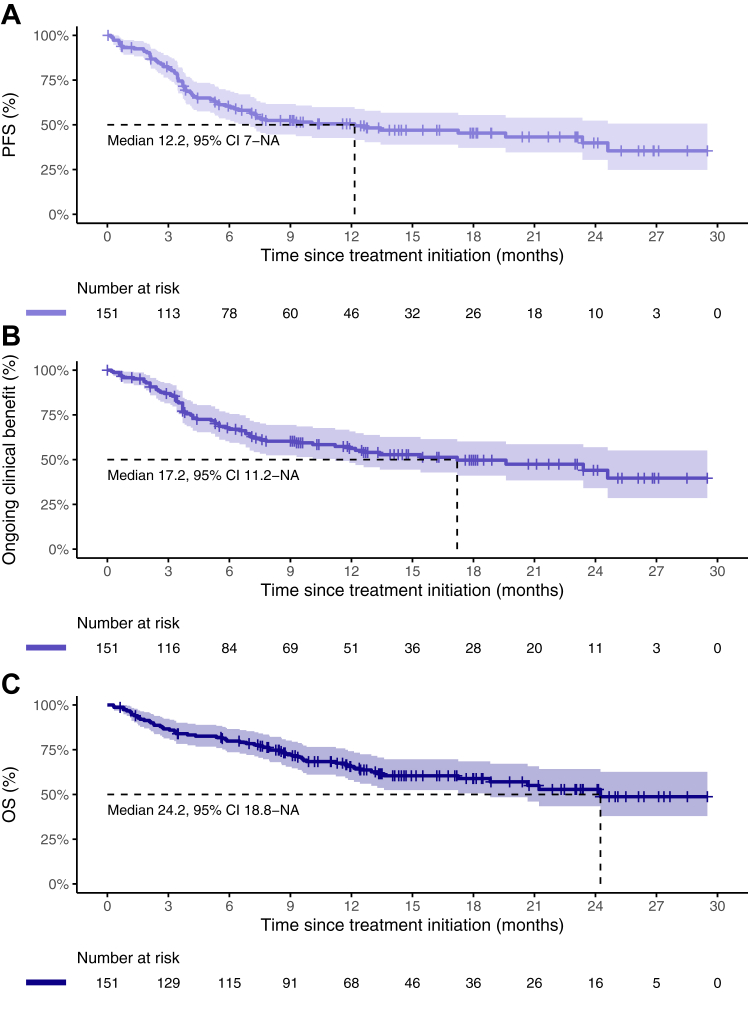
**A–C: Progression-free survival, time to end of clinical benefit and overall survival.** Legend: Kaplan–Meier curves for estimated progression-free survival (3A), time to end of clinical benefit (3B), and overall survival (3C), with 95% confidence intervals (shading). Median survival is indicated with dashed lines and annotated in the figures. Abbreviations: PFS, progression-free survival; OS, overall survival; CI, confidence interval; NA, not available.

The median time on treatment was 26.0 weeks (95% CI, 21.3–32.1) (Fig. 3). Both for confirmed objective responders and for all objective responders, the median DoR had not been reached at data cut-off. At 24 months after the first response was measured, 65.2% (95% CI, 41.0–100) of confirmed responders and 58.0% (95% CI, 36.1–93.2) of all responders had an ongoing response. The median PFS was 12.2 months (95% CI, 7.0-not reached (NR)) and the median OS was 24.2 months (95% CI, 18.8-NR), at a median duration of follow-up of 15.2 months (Fig. 4A and C). The PFS and OS probability at 24 months were 39.9% (95% CI, 40.4–52.4) and 52.8% (95% CI, 43.4–64.2), respectively. The median time to end of CB was 17.2 months (95% CI, 11.2-NR, Fig. 4B). At data cut-off, 14 patients were still on treatment. Another 44 patients (29.1%) were on a treatment pause after at least 24 weeks of treatment with ongoing CB (Fig. 3). Among these, two patients experienced PD at 6.8 and 12.8 months. Neither of them resumed cemiplimab treatment. The probability of remaining progression-free after 12 months and 18 months of treatment interruption were 94.1% (95% CI, 83.6–100) and 80.7% (95% CI, 58.3–100), respectively. The main reason for permanent treatment discontinuation was disease progression (54 of 93 patients that stopped treatment, 58.1%, Supplementary Fig. S1).

### Safety

A total of 68 AEs of grade ≥ 3 occurred in 45 of 151 patients (29.8%). Of all grade ≥ 3 AEs, 42 (61.8%) were TREAs (Table 4), which occurred in 28 of 151 patients (18.5%). Of note, the most commonly reported TRAE was rejection of a kidney transplant (n = 4, 9.5%). All four of these patients required an allograft nephrectomy and started with dialysis. Other commonly reported TRAEs were hypertension (n = 3, 4.8%) and increased gamma glutamyl transferase (n = 3, 4.8%). Fatal AEs occurred in three patients (2%); one patient unexpectedly passed away in his sleep from an unknown cause, which was deemed not related to treatment. Another event concerned a patient with grade 4 neutropenia, possibly related to treatment. However, this patient also had chronic lymphocytic leukemia, which probably contributed to the AE as a consequence of bone marrow infiltration. Cemiplimab was discontinued and the patient was treated with high doses of steroids, but deceased from this event. The last fatal AE concerned a patient with grade 5 delusions, possibly related to treatment. Potential underlying causes of the delusions were hypocalcaemia, an infection, or an immune-related encephalitis. The patient was subsequently treated with calcium suppletion and steroids, but deteriorated rapidly and died. In total, 15 patients (9.9%) discontinued treatment due to an AE. No suspected unexpected serious adverse reactions occurred.

**Table 4:** Possibly, probably, or definitely related adverse events grade ≥ 3.

Adverse events	Grade 3	Grade 4	Grade 5
Alanine aminostransferase increased	1	0	0
Alkaline phosphatase increased	1	0	0
Anemia	1	0	0
Aspartate aminotransferase increased	1	0	0
Colitis	2	0	0
Creatinine increased	1	0	0
Delusions	0	0	1
Diarrhea	2	0	0
Dizziness	1	0	0
Dyspnea	1	0	0
Fatique	1	0	0
Fever	1	0	0
Gamma glutamyltransferase increased	3	0	0
Hepatitis	1	0	0
Hypercalcemia	0	2	0
Hypertension	3	0	0
Hyperthyroidism	1	0	0
Hypophysitis	1	0	0
Hypotension	1	0	0
Interstitial nephritis	1	0	0
Kidney transplant rejection^a^	2	2	0
Lipase increased	0	1	0
Nephrotic syndrome	1	0	0
Neutrophil count decreased	0	1	0
Pancreatitis	1	0	0
Pneumonitis	1	0	0
Pruritus	2	0	0
Rash maculo-papular	1	0	0
Sepsis	0	1	0
Skin toxicity not otherwise specified	1	0	0
Vomiting	1	0	0

a These were originally reported as acute kidney injuries.

## Discussion

To the best of our knowledge, this is the first prospective real-world protocol assessing efficacy and safety of PD-1 blocker cemiplimab in patients with laCSCC not amendable to curative surgery or radiotherapy, or mCSCC. In our cohort, cemiplimab demonstrated an objective CBR of 54.3% (95% CI, 46.0–62.4), with an ORR of 35.1% (95% CI, 27.5–43.3). Median PFS and OS were 12.2 months (95% CI, 7.0-NR) and 24.2 months (95% CI, 18.8-NR), respectively. Additionally, the physician-assessed CBR was 59.6% (95% CI, 51.3–67.5), with the median time until the end of physician-assessed CB being 17.2 months (95% CI, 11.2-NR). All aforementioned findings point towards durable benefit in a significant proportion of patients.

Although the outcomes observed in our cohort are comparable to previous retrospective real-world studies,^17^, ^18^, ^19^, ^20^, ^21^, ^22^ some are more modest compared to the registration trials.^4^^,^^7^^,^^12^ While the observed ORR of 35.1% obtained in this cohort might appear slightly lower than the ORRs of approximately 45% in previous phase II trials, it is important to note that the point estimate of our cohort falls within the 95% CI of the ORRs reported in those trials (32–55%).^4^^,^^7^ The potential difference in true ORR is probably inherent to the real-world setting of DAP. For instance, a significant proportion of patients (n = 33, 21.8%) in this cohort did not have measurable disease according to RECIST v1.1 criteria at baseline and hence, could not be evaluated for PR. When evaluating only patients with measurable disease, the ORR was 41%, which more closely resembles the point estimate of the phase II ORRs.^4^^,^^7^ However, the 60.6% (95% CI, 53.3–67.6)^4^^,^^7^^,^^12^ DCR at 15 weeks found in the phase II trials is slightly higher than our 54.3% (95% CI, 46.0–62.4) CBR at 16 weeks and we observed a lower median PFS (12.2 months vs 18.4 months) and 24-months OS probability (52.8% vs 73.3%) compared to the previous phase II trials.^12^

These disparate outcomes may be attributed to specific patient characteristics within this real-world setting. To illustrate, the median age of our cohort was 78 years compared to 72 years in registration trials. Furthermore, in contrast to the registration trials, we enrolled patients with a poor performance status (ECOG ≥ 2), constituting 13.3% of our cohort, as well as a small number of patients with autoimmune diseases (n = 17, 11.3%), solid organ transplantations (n = 7, 4.6%), or hematological malignancies (n = 13, 8.6%). Whereas autoimmune diseases or (often associated) use of corticosteroids or other immunosuppressive drugs were not significantly associated with clinical outcomes, both higher ECOG and the presence of hematological malignancies were significantly associated with a lack of CB. Yet, durable objective responses were still observed in both subgroups. Moreover, among the seven patients with a history of kidney transplant, four (57.1%) patients experienced a kidney transplant rejection—aligning with the rate of acute kidney transplant rejection after ICB treatment described in literature (42%)^23^—but all four of these patients also demonstrated CB according to their treating physician. Considering the implications of these findings on the eligibility criteria for cemiplimab treatment in daily clinical practice, there may be a rationale for extending this therapeutic option to patients with a history of autoimmune disease. In contrast, caution might be warranted in treating patients with ECOG ≥ 2 or hematological malignancies, given their association with a decreased chance of CB. However, due to the limited patient numbers, our data are insufficient to fully exclude these patients from this potentially effective treatment. For the organ transplant patients, who are at 65–250 times higher risk of CSCC due to chronic use of immunosuppressive agents,^6^^,^^7^ a deeper understanding of the risk factors for acute rejection is essential. In the meantime, treatment with cemiplimab may still be considered, provided that patients are properly informed and advantages and disadvantages (especially the high likelihood of acute transplant rejection) are carefully weighed.

Besides eligibility, the procedure of response evaluations in our cohort also differed from the registration trials.^4^^,^^7^ Firstly, response evaluations in our cohort were performed at 16 weeks and every 12 weeks thereafter, as opposed to every 8 weeks. Secondly, besides traditional RECIST v1.1 measurements, physician-assessed CB based on all available clinical parameters was used as an efficacy outcome to guide clinical decision-making and give a more accurate representation of the therapeutic benefit of cemiplimab in daily clinical practice. This outcome measure was included because solely radiological evaluation of CSCC is challenged by its superficial location, as is reflected by the large proportion of patients in our cohort that did not have measurable disease (21.9%) or was not evaluable (13.2%). Additionally, hereby patients that technically fulfill the requirements for PD, for instance due to a newly enlarged lymph node with otherwise good tumor response, could still be considered to have CB as appropriate. Given that the current treatment guidelines for cemiplimab in CSCC lack standardized response evaluation protocols,^24^ our data support the rationale for a 12-week interval for response evaluations, guided by physician assessments incorporating both radiological and clinical parameters. Notably, the pragmatic, physician-assessed CBR showed limited discordance with the objective CBR in our cohort (5.3%) and was highly consistent with other real-world studies (59.6% in our cohort vs 59.6% demonstrated in 240 patients by Hober et al.^18^). Moreover, the appropriateness of physician-assessed outcomes in a real-world setting is underscored by their close alignment with trial outcomes, exemplified by our physician-assessed CBR of 59.6% (95% CI, 51.3–67.5) compared to their DCR of 60.6% (95% CI, 53.3–67.6)^4^^,^^7^^,^^12^ and our median 17.2 months to end of CB vs their 18.4 months PFS.^12^

Lastly, our data provide novel insights into early treatment discontinuation. Current guidelines recommend to stop cemiplimab after 24 months of treatment or earlier in case of PD.^25^ However, in our cohort, interruption of treatment in case of ongoing benefit was permitted after a minimum of six months of treatment. Forty-four patients (29.1%) in our cohort stopped treatment for this reason. In this group, the probability of remaining progression-free 18 months later was 80.7% (95% CI, 58.3–100). Considering the potential benefits of early treatment discontinuation, including preventing overtreatment, minimizing the risk of potential immune-related AEs, and promoting sustainable healthcare, these data show that discontinuation of cemiplimab upon durable benefit may be a safe approach and can be considered after six months. Yet, the follow-up data of these patients after treatment discontinuation are currently too immature to make a formal recommendation.

Limitations of this protocol include the lack of a control arm. However, given the significant treatment effect seen in previous phase II trials of cemiplimab in laCSCC and mCSCC,^4^^,^^7^^,^^12^ conducting randomized trials in this setting is deemed unethical. Furthermore, results from real-world studies tend to be more modest, as these studies are conducted in less controlled circumstances than in traditional clinical trials. This inherent difference should be taken into consideration when making a direct comparison between the two. Nevertheless, collecting prospective RWD on the effectivity and safety of anti-cancer drugs, also in an older, more frail patient population with substantial comorbidities is crucial to generate realistic data on drug performance in daily clinical practice.

The real-world effectiveness and safety data gathered within this platform provided additional evidence for a positive final health technology assessment in the Netherlands. This illustrates that an initiative like DAP can help to bridge the gap between drug approval by regulatory agencies and reimbursement on a national level. DAP is beneficial for patients, as it provides accelerated access to promising anti-cancer drugs, but also for manufacturers and payers, as the personalized reimbursement model addresses financial risk collaboratively, thereby supporting sustainable healthcare. As the reliance on single-arm studies increases, the demand for demonstrating the real-world value of novel anti-cancer drugs will expand, in which innovative protocols such as DAP may facilitate.

In conclusion, DAP provided patients with laCSCC or mCSCC early access to PD-1 blocker cemiplimab through a personalized reimbursement scheme. The data generated within this protocol contributed to the reimbursement of cemiplimab in the Netherlands, provided novel insights into the effectiveness of cemiplimab across various real-world subpopulations, offered potential pragmatic methods for treatment evaluations, and explored early treatment discontinuation possibilities.

## Contributors

Karlijn Verkerk and Birgit S. Geurts contributed with conceptualization, resources, data curation, formal analysis, investigation, writing-original draft, and project administration. Laurien J. Zeverijn contributed with resources, data curation, investigation, project administration, and writing review and editing. Vincent van der Noort contributed with formal analysis and writing review and editing. Henk M.W. Verheul contributed with writing review and editing. John B.A.G. van Haanen, Astrid A.M. van der Veldt, Ferry A.L.M. Eskens, Maureen J.B. Aarts, Carla M.L van Herpen, Mathilde Jalving, Jourik A. Gietema, Lot. A. Devriese, Mariette Labots, and Sahar Barjesteh van Waalwijk van Doorn-Khosrovani contributed with investigation, resources, and writing review and editing. Egbert F. smit and Haiko J. Bloemendal were both principle investigators and contributed with resources, supervision, funding acquisition, investigation, writing original draft, project administration, and writing review and editing.

## Data sharing statement

The data supporting the findings of this study are available within the paper and its Supplementary materials. Additionally, the deidentified raw data required to reproduce the reported results are available upon reasonable request from the corresponding author Prof. H. J. Bloemendal, at Haiko.Bloemendal@radboudumc.nl. We are committed to promoting transparency and reproducibility in scientific research and encourage fellow researchers to reach out for access to the data for further analysis and validation.

## Declaration of interests

John B.A.G. Haanen reports research grants to the institution from Asher Bio, Amgen, BioNTech, Bristol Myers Squibb, Novartis, and Sastra Cell Therapy, consultation fees to the institution from Achilles Tx, AstraZeneca, BioNTech, Bristol Myers Squibb, CureVac, Eisai, GlaxoSmithKline, Imcyse, Immunocore, Instil Bio, Iovance Bio, Merck, Merck Sharp & Dohme, Neogene Tx, Novartis, Obsidian Tx, Roche, Sanofi, Sastra Cell Therapy, Third Rock Ventures, and T-Knife, and stock options from Neogene Tx and Sastra Cell Therapy. Mathilde Jalving received compensation to the institution for advisory roles for Pierre Fabre, Merck, and Novartis. Lot A. Devriese received compensation to the institution for advisory roles and education for Bristol Myers Squibb, Merck Sharp & Dohme, and Incyte. Astrid A.M. van der Veldt received compensation to the institution for advisory roles for Bristol Myers Squibb, Merck Sharp & Dohme, Eisai, Ipsen, Novartis, Pierre Fabre, Pfizer, Roche, and Sanofi. Maureen J.B. Aarts reports research grants to the institution from Merck-Pfizer and consultation fees to the institution from being on the advisory board from Amgen, Bristol Myers Squibb, Novartis, Merck Sharp & Dohme, Merck-Pfizer, Pierre Fabre, Sanofi, Astellas, and Bayer. Carla M.L. van Herpen reports research grants to the institution from Astra Zeneca, Bayer, Bristol Myers Squibb, Elevar, Ipsen, Merck Sharp & Dohme, Merck, Novartis, and Sanofi, payments for lectures to the institution from ICCR, NIFU, RIVM, Roije Congressen, Nederlands Paramedisch Instituut, Elevar, and Uitgeverij Jaap, and payments to the institution for participating on an advisory board to the institution from Elevar. Mariette Labots received compensation to the institution for education for Bristol Myers Squibb and Janssen. Sahar Barjesteh van Waalwijk van Doorn-Khosrovani reports the PRIME-ROSE—A European precision cancer medicine trial network and implementation initiative funded by the EU Cancer Mission; grant no. 101104269 paid to the Leiden University Medical Center and travel expenses for attending meetings at the Nordic Precision Medicine Forum and Center for Innovation in Regulatory Science. Egbert F. Smit reports compensation to the institution for advisory roles at AstraZeneca, Bristol Myers Squibb, Boehringer Ingelheim, Daiichi Sankyo, Eli Lilly, Janssen, Merck Sharp & Dohme, Roche, Sanofi, and Takeda, personal compensation for education from Boehringer Ingelheim and Daiichi Sankyo, and compensation to the institution for participation in the data safety monitoring board of DSI. The other authors have no conflicts of interests to disclose.

## Appendix A Supplementary data

Supplementary Fig. S1 and Table S1A–C
