# Supplementary material for: Cemiplimab in locally advanced or metastatic cutaneous squamous cell carcinoma: prospective real-world data from the DRUG Access Protocol
Source: Lancet Reg Health Eur. 2024 Mar 5;39:100875. doi: 10.1016/j.lanepe.2024.100875 (PMC10924203; doi:10.1016/j.lanepe.2024.100875)
Supplement: Supplementary Fig. S1 and Table S1A–C [file mmc1.pdf]

Supplementary materials

Contents

Supplementary Tables 2
Supplementary Table 1A. Baseline characteristics stratified by objective clinical benefit at 16 weeks 2
Supplementary Table 1B. Baseline characteristics stratified by physician-assessed clinical benefit at 16 weeks 3
Supplementary Table 1C. Baseline characteristics of patients with measurable disease at baseline stratified by objective response 4
Supplementary Figures 5
Supplementary Figure 1. CONSORT flow diagram 5

Supplementary Tables

Supplementary Table 1A. Baseline characteristics stratified by objective clinical benefit at 16 weeks

	**Clinical benefit** n = 82	**No clinical benefit** n = 69	**P value**
**Age at consent, median [IQR]**	79.0 [76.0, 82.75]	76.0 [68.0, 84.0]	1.000
**Biological sex, n (%)**			0.134
Male	56 (68.3)	48 (69.6)	
Female	26 (31.7)	21 (30.4)	
**ECOG performance status, n (%)**			0.043
ECOG 0	26 (31.7)	19 (27.5)	
ECOG 1	49 (59.8)	36 (52.2)	
ECOG 2	5 (6.1)	14 (20.3)	
ECOG 3	1 (1.2)	0 (0.0)	
Not Available	1 (1.2)	0 (0.0)	
**Primary site, n (%)**			0.121
Head or neck	57 (69.5)	35 (50.7)	
Trunk	11 (13.4)	13 (18.8)	
Upper or lower extremities	11 (13.4)	12 (17.4)	
Other[a]	1 (1.2)	5 (7.2)	
More than one site[b]	1 (1.2)	3 (4.3)	
Unknown	1 (1.2)	1 (1.4)	
**Type, n (%)**			0.864
mCSCC	53 (64.6)	46 (66.7)	
laCSCC	29 (35.4)	23 (33.3)	
**Previous systemic treatment, n (%)**			0.143
No	80 (97.6)	63 (91.3)	
Yes	2 (2.4)	6 (8.7)	
**Previous radiotherapy, n (%)**			0.105
Yes	48 (58.5)	31 (44.9)	
No	34 (41.5)	38 (55.1)	
**History of autoimmunedisease, n (%)**			0.609
No	74 (90.2)	60 (87.0)	
Yes	8 (9.8)	9 (13.0)	
**Corticosteroid use, n (%)**			1.000
No	76 (92.7)	64 (92.8)	
Yes	6 (7.3)	5 (7.2)	
**Other immunosuppressive drugs, n (%)**			0.070
No	79 (96.3)	65 (94.2)	
Yes	3 (3.7)	4 (5.8)	
**History of organ transplantation, n (%)**			0.070
No	79 (96.3)	65 (94.2)	
Yes	3 (3.7)	4 (5.8)	
**History of hematological malignancy, n (%)**			0.022
No	79 (96.3)	59 (85.5)	
Yes	3 (3.7)	10 (14.5)	

IQR, interquartile range; ECOG, Eastern Cooperative Oncology Group; laCSCC, locally advanced cutaneous squamous cell carcinoma; mCSCC, metastatic cutaneous squamous cell carcinoma.

[a] Tumor location of patient with clinical benefit included feet (n=1). Tumor locations of patients without clinical benefit included conjunctiva (n=2), hand right (n=1), forefinger right (n=1), and feet (n=1).

[b] Tumor locations of patients with clinical benefit included ear, clavicular, and scalp (n=1). Tumor locations of patients without clinical benefit included head, trunk and arms (n=1), both legs and left arm (n=1), and head and trunk (n=1).

Supplementary Table 1B. Baseline characteristics stratified by physician-assessed clinical benefit at 16 weeks

	**Clinical benefit** n = 90	**No clinical benefit** n = 61	**P value**
**Age at consent, median [IQR]**	78.50 [74.3, 82.0]	78.0 [72.0, 84.0]	1.000
**Biological sex, n (%)**			0.858
Male	62 (68.9)	42 (68.9)	
Female	28 (31.1)	19 (31.1)	
**ECOG performance status, n (%)**			0.012
ECOG 0	30 (33.3)	15 (24.6)	
ECOG 1	53 (58.9)	32 (52.5)	
ECOG 2	5 (5.6)	14 (23.0)	
ECOG 3	1 (1.1)	0 (0.0)	
Not Available	1 (1.1)	0 (0.0)	
**Primary site, n (%)**			0.210
Head or neck	60 (66.7)	32 (52.5)	
Trunk	14 (15.6)	10 (16.4)	
Upper or lower extremities	12 (13.3)	11 (18.0)	
Other[a]	1 (1.1)	5 (8.2)	
More than one site[b]	2 (2.2)	2 (3.3)	
Unknown	1 (1.1)	1 (1.6)	
**Type, n (%)**			1.000
mCSCC	59 (65.6)	40 (65.6)	
laCSCC	31 (34.4)	21 (34.3)	
**Previous systemic treatment, n (%)**			0.062
No	88 (97.8)	55 (90.2)	
Yes	2 (2.2)	6 (9.8)	
**Previous radiotherapy, n (%)**			0.135
Yes	52 (57.8)	27 (44.3)	
No	38 (42.2)	34 (55.7)	
**History of autoimmunedisease, n (%)**			0.605
No	81 (90.0)	53 (86.9)	
Yes	9 (10.0)	8 (13.1)	
**Corticosteroid use, n (%)**			0.526
No	82 (91.1)	58 (95.1)	
Yes	8 (8.9)	3 (4.9)	
**Other immunosuppressive drugs, n (%)**			0.702
No	85 (94.4)	59 (96.7)	
Yes	5 (5.6)	2 (3.3)	
**History of organ transplantation, n (%)**			0.702
No	85 (94.4)	59 (96.7)	
Yes	5 (5.6)	2 (3.3)	
**History of hematological malignancy, n (%)**			0.007
No	87 (96.7)	51 (83.6)	
Yes	3 (3.3)	10 (16.4)	

IQR, interquartile range; ECOG, Eastern Cooperative Oncology Group; laCSCC, locally advanced cutaneous squamous cell carcinoma; mCSCC, metastatic cutaneous squamous cell carcinoma.

[a] Tumor location of patient with clinical benefit included feet (n=1). Tumor locations of patients without clinical benefit included conjunctiva (n=2), hand right (n=1), forefinger right (n=1), and feet (n=1).

[b] Tumor locations of patients with clinical benefit included ear, clavicular and scalp (n=1), and head, trunk and arms (n=1). Tumor locations of patients without clinical benefit included both legs and left arm (n=1) and head and trunk (n=1).

Supplementary Table 1C. Baseline characteristics of patients with measurable disease at baseline stratified by objective response

	Objective response n = 48	No objective response n = 70	P value
**Age at consent, median [IQR]**	79.00 [75.75, 83.25]	76.00 [69.00, 83.00]	0.227
**Biological sex, n (%)**			1.000
Male	33 (68.8)	47 (67.1)	
Female	15 (31.2)	23 (32.9)	
**ECOG performance status, n (%)**			0.076
ECOG 0	15 (31.2)	18 (25.7)	
ECOG 1	29 (60.4)	36 (51.4)	
ECOG 2	3 (6.2)	15 (21.4)	
ECOG 3	0 (0.0)	1 (1.4)	
Not Available	1 (2.1)	0 (0.0)	
**Primary site, n (%)**			0.032
Head or neck	36 (75.0)	34 (48.6)	
Trunk	8 (16.7)	13 (18.6)	
Upper or lower extremities	3 (6.2)	15 (21.4)	
Other[a]	1 (2.1)	5 (7.1)	
More than one site[b]	0 (0.0)	1 (1.4)	
Unknown	0 (0.0)	2 (2.9)	
**Type, n (%)**			1.000
mCSCC	32 (66.7)	47 (67.1)	
laCSCC	16 (33.3)	23 (32.9)	
**Previous systemic treatment, n (%)**			0.647
No	47 (97.9)	66 (94.3)	
Yes	1 (2.1)	4 (5.7)	
**Previous radiotherapy, n (%)**			0.189
Yes	28 (58.3)	31 (44.3)	
No	20 (41.7)	39 (55.7)	
**History of autoimmunedisease, n (%)**			0.759
No	44 (91.7)	62 (88.6)	
Yes	4 (8.3)	8 (11.4)	
**Corticosteroid use, n (%)**			1.000
No	45 (93.8)	65 (92.9)	
Yes	3 (6.2)	5 (7.1)	
**Other immunosuppressive drugs, n (%)**			1.000
No	46 (95.8)	67 (95.7)	
Yes	2 (4.2)	3 (4.3)	
**History of organ transplantation, n (%)**			1.000
No	46 (95.8)	66 (94.3)	
Yes	2 (4.2)	4 (5.7)	
**History of hematological malignancy, n (%)**			0.470
No	46 (95.8)	64 (91.4)	
Yes	2 (4.2)	6 (8.6)	

IQR, interquartile range; ECOG, Eastern Cooperative Oncology Group; laCSCC, locally advanced cutaneous squamous cell carcinoma; mCSCC, metastatic cutaneous squamous cell carcinoma.

[a] Tumor location of patient with objective response included feet (n=1). Tumor locations of patients without objective response included conjunctiva (n=2), hand right (n=1), forefinger right (n=1), and feet (n=1).

[b] Tumor locations of patients without objective response included both legs and left arm (n=1).

Supplementary Figures

Supplementary Figure 1. CONSORT flow diagram

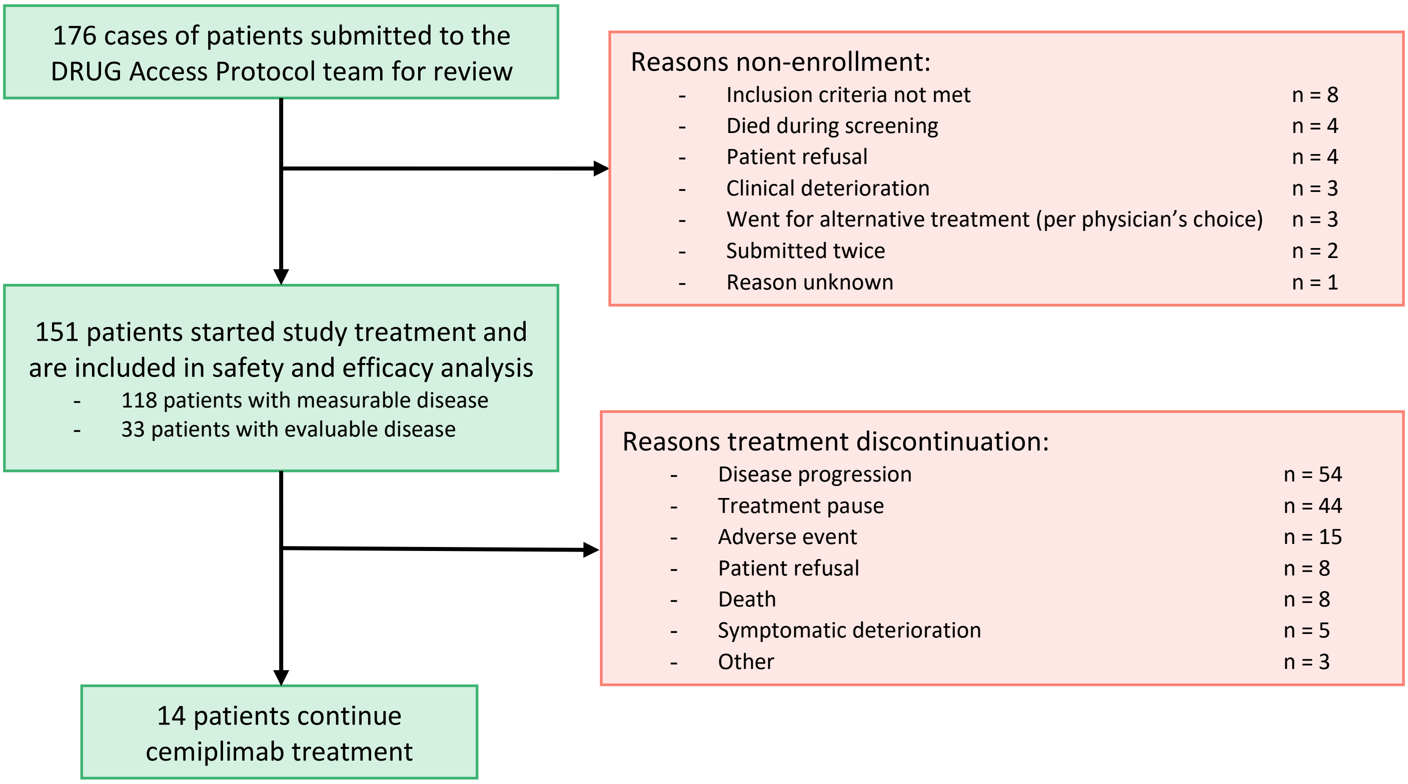

Legend: CONSORT flow diagram of patients with locally advanced or metastatic squamous cell carcinoma submitted to DRUG Access Protocol team between February 2021 and December 2022, and reasons for non-enrollment and end of treatment.
